# Outcomes of Combined Therapy With Focal Laser and Intravitreal Bevacizumab for Retinal Arterial Macroaneurysm: A Case Series

**DOI:** 10.7759/cureus.81382

**Published:** 2025-03-28

**Authors:** Alex Jemelian, Moises Enghelberg

**Affiliations:** 1 Department of Vitreoretinal Diseases and Surgery, Dougherty Laser Vision (DLV) Vision, Los Angeles, USA; 2 Department of Retina Service, Loma Linda University Eye Institute, Loma Linda, USA

**Keywords:** anti-vascular endothelial growth factor, focal laser, intravitreal bevacizumab, laser therapy, macular edema, retinal macroaneurysm, retinal vasculature, retina surgery

## Abstract

Retinal arterial macroaneurysms (RAMAs) are uncommon vascular abnormalities that can lead to significant visual impairment if left untreated. While various management strategies exist, there is no universally accepted standard of care, and treatment is often guided by the severity of associated hemorrhage and fluid accumulation.

The purpose of this case series is to evaluate and discuss the management and the use of combination therapy in the form of focal laser (FL) treatment and intravitreal anti-vascular endothelial growth factor (VEGF) injections in patients with retinal arterial macroaneurysms (RAMAs). A retrospective chart review was conducted on the patients, including their clinical histories, comorbidities, age, and treatment methods. Baseline and posttreatment vision were obtained, and a detailed literature review was conducted emphasizing treatment and outcomes. All three patients had a complete resolution of the subretinal and intraretinal fluid associated with the RAMA with combination therapy with considerable improvement in vision. The average improvement in visual acuity (VA) across the three cases was 0.55 logarithm of the minimum angle of resolution (logMAR). The central retinal thickness (CRT) decreased on average by 275.7 µm at the three-month mark. Combination therapy with anti-VEGF and focal laser appears to be an effective and definitive treatment for retinal arterial macroaneurysms.

## Introduction

Retinal arterial macroaneurysms (RAMAs) are small bulges that form in the walls of retinal arteries due to structural weakness in the muscular layer [1-5]. This structural weakness is often caused by chronic conditions, which include hypertension, diabetes, arteriosclerosis, and rheumatoid conditions. These chronic conditions place excessive stress on the vascular walls over time. As the arterial walls weaken, the arterial muscular wall bulges outward, forming a balloon-like structure known as a macroaneurysm [1,4]. These macroaneurysms are fragile and can rupture, consequently leaking blood into the surrounding retinal tissue [5].

A case-control study analyzed the pathophysiology and systemic risk factors of RAMA in 43 patients with 52 confirmed cases of RAMA and compared them to 43 patients without RAMA [6]. A trend was found showing that RAMA was more common in female patients, in those with retinal vein occlusions and hypertension. In the analysis, patients with RAMA were at a higher likelihood of having lower visual acuity (VA) [6]. RAMA more commonly occurs near the macula, leading to severe visual deterioration and diminished visual acuity. Without intervention, the fluid accumulation and resultant tissue damage can lead to permanent vision loss. There is no consensus or standardized treatment approach to RAMAs [3]. Treatments such as focal laser (FL) therapy or intravitreal injections of anti-vascular endothelial growth factor (VEGF) aim to mitigate these effects by stabilizing the compromised vessel and preventing further retinal injury [2,3,7-13]. The anti-VEGF injection utilized for our study is bevacizumab, a monoclonal antibody that inhibits VEGF to prevent abnormal blood vessel growth and leakage in retinal conditions. By blocking VEGF, bevacizumab helps stabilize the retinal vasculature and reduce retinal swelling, which can lead to improved visual outcomes in patients with RAMA [9,11,12].

The purpose of this report is to further discuss the management and the use of combination therapy and its synergistic effect in the form of focal laser and intravitreal anti-VEGF injections in patients with RAMA.

## Case presentation

Case 1

**Figure 1:**
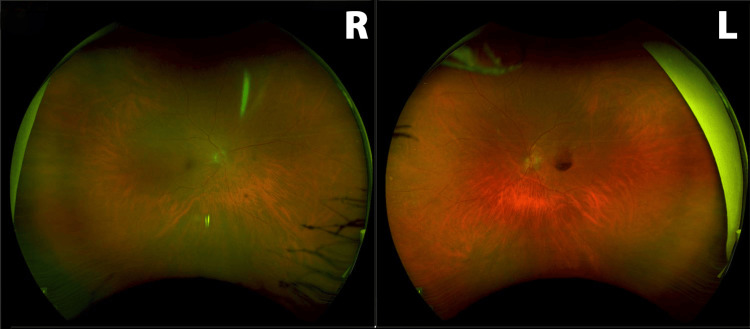
Case 1 Fundus Photograph

**Figure 2:**
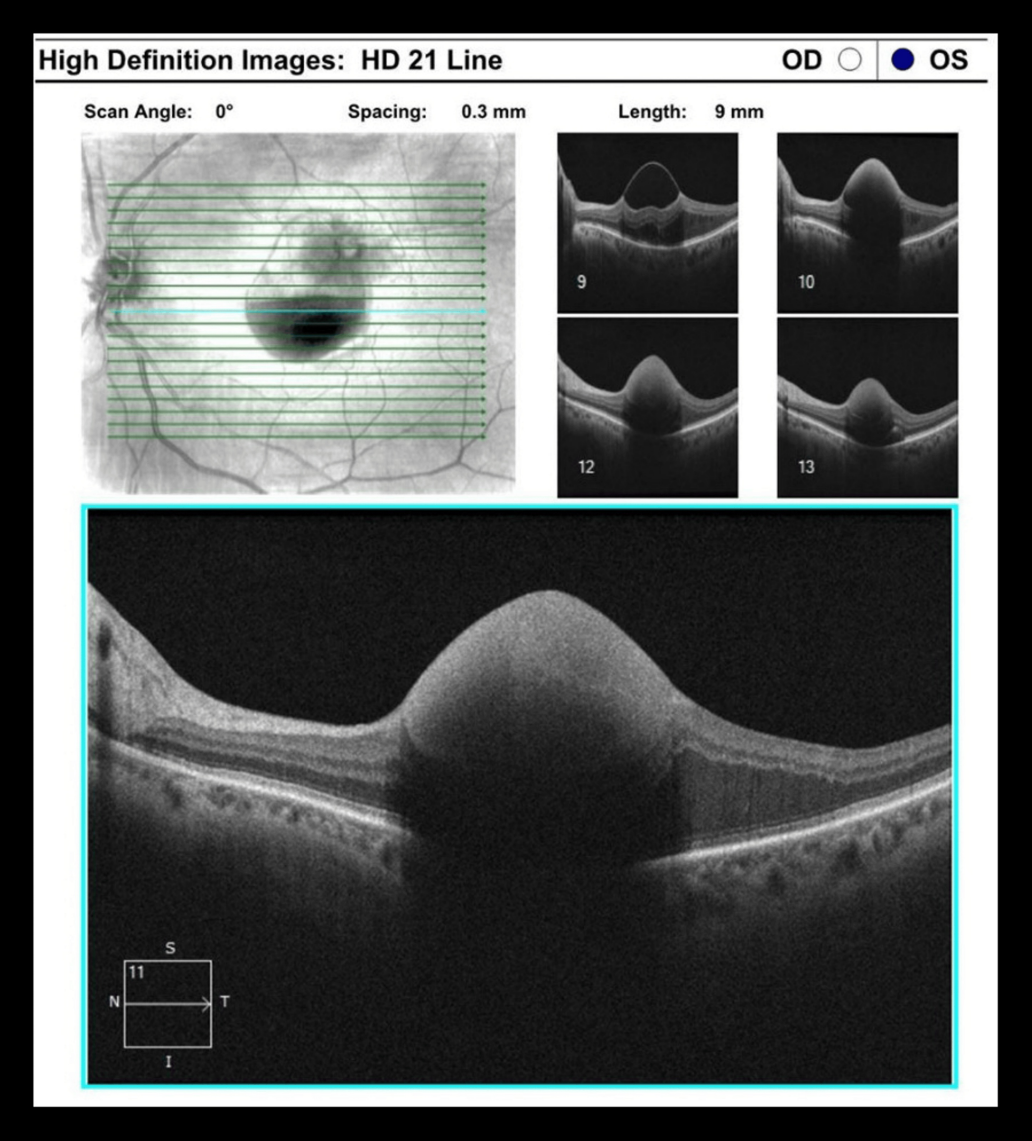
Case 1 OCT Scan Prior to Treatment OCT: optical coherence tomography

**Figure 3:**
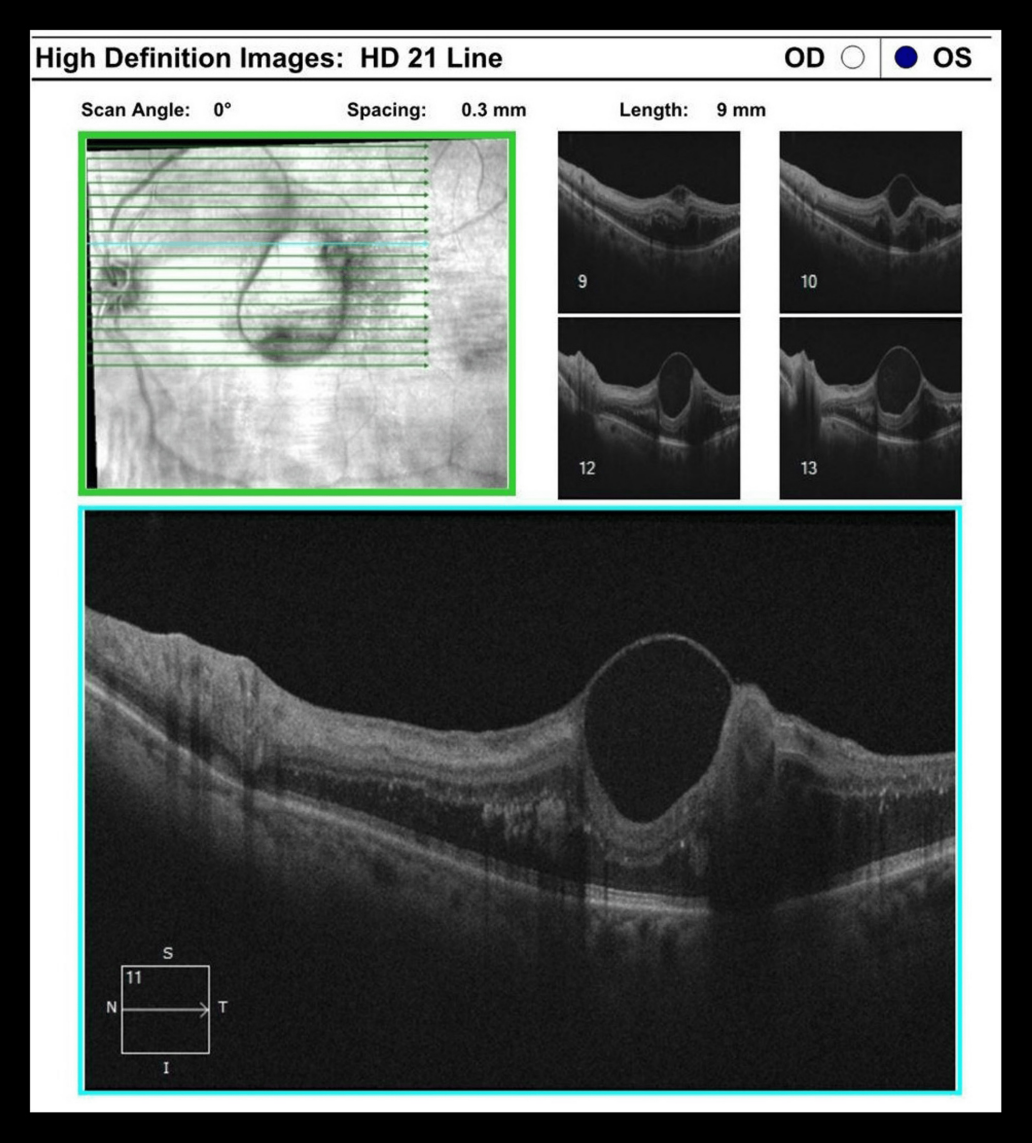
Case 1 OCT Scan at Six-Week Follow-Up OCT: optical coherence tomography

**Figure 4:**
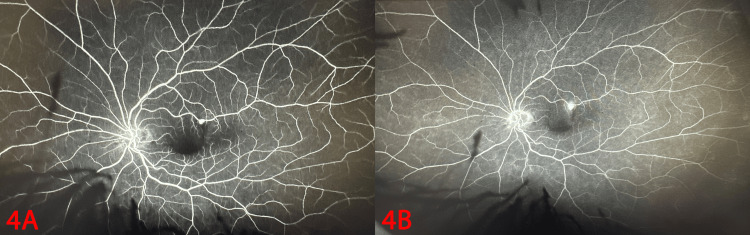
Case 1 Fluorescein Angiography

The patient is a 78-year-old Caucasian woman with a past medical history of hypertension. Presenting with 20/25 right eye (OD) and "hand motion" vision left eye (OS), a retinal arterial macroaneurysm was noted on the superior arcade with subhyaloid hemorrhage and blocking of the fovea (Figures 1, 2). The need for intravitreal injections and focal lasers to the RAMA was discussed with the patient. The patient received bevacizumab 1.25 mg/0.05 mL every month for three consecutive months. One week after the first intravitreal dose of bevacizumab, she underwent a focal laser (Iridex IQ 532™ Dual Port Green Laser System, Mountain View, CA) with the following settings: 50 µm spot size, 150 mW power, and 140 ms duration. A laser was applied and titrated until blanching of the RAMA was noted; the accompanying area of thickening was not addressed with the laser. At the six-week follow-up, an optical coherence tomography (OCT) was obtained evidencing that the hemorrhage had begun to disperse from the foveola (Figure 3). Fluorescein angiography was performed, demonstrating proper flow distal to the aneurysm (Figure 4A, 4B). Vision at the three-month follow-up visit was 20/200 OS.

Case 2

**Figure 5:**
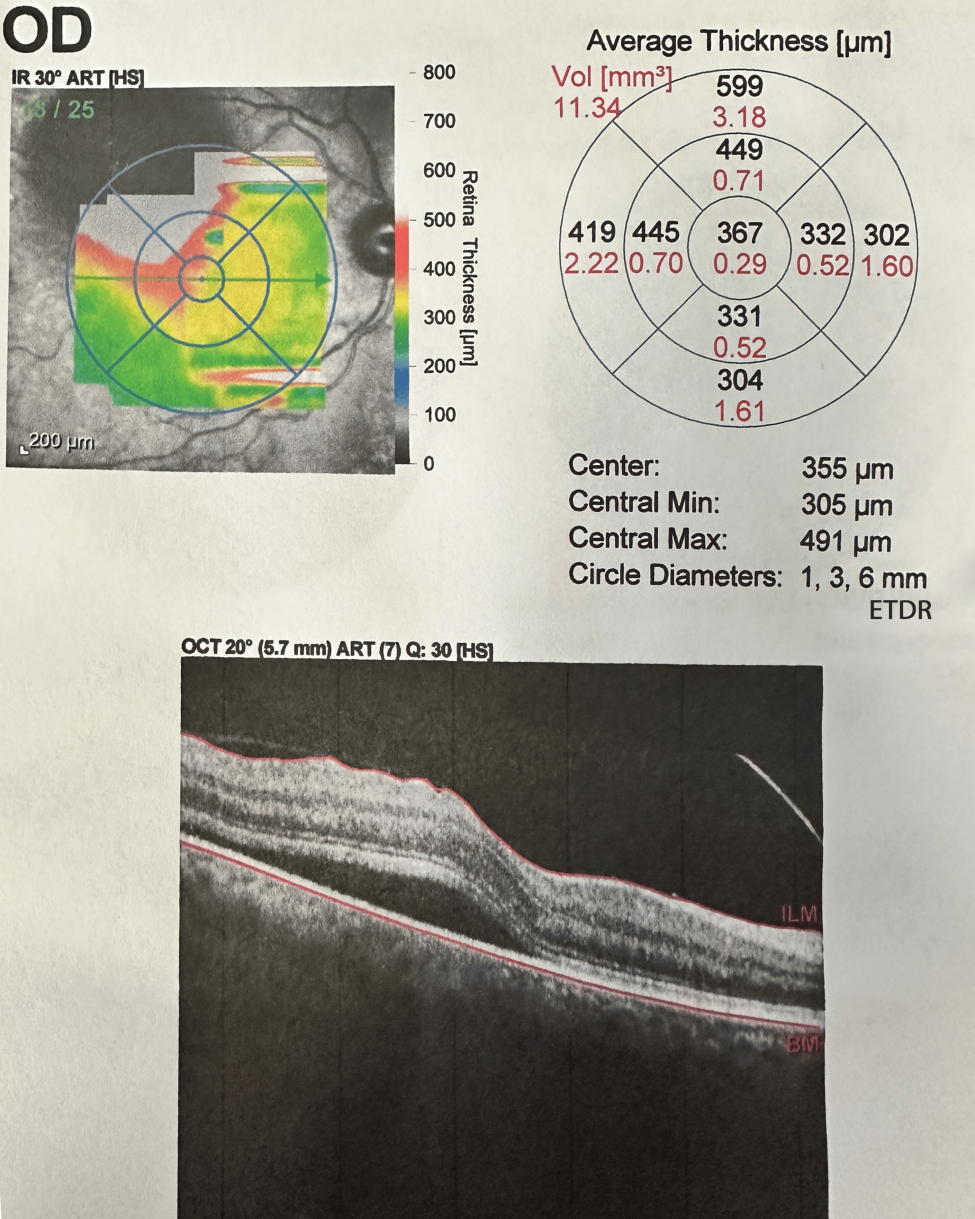
Case 2 OCT Scan Prior to Treatment OCT: optical coherence tomography

**Figure 6:**
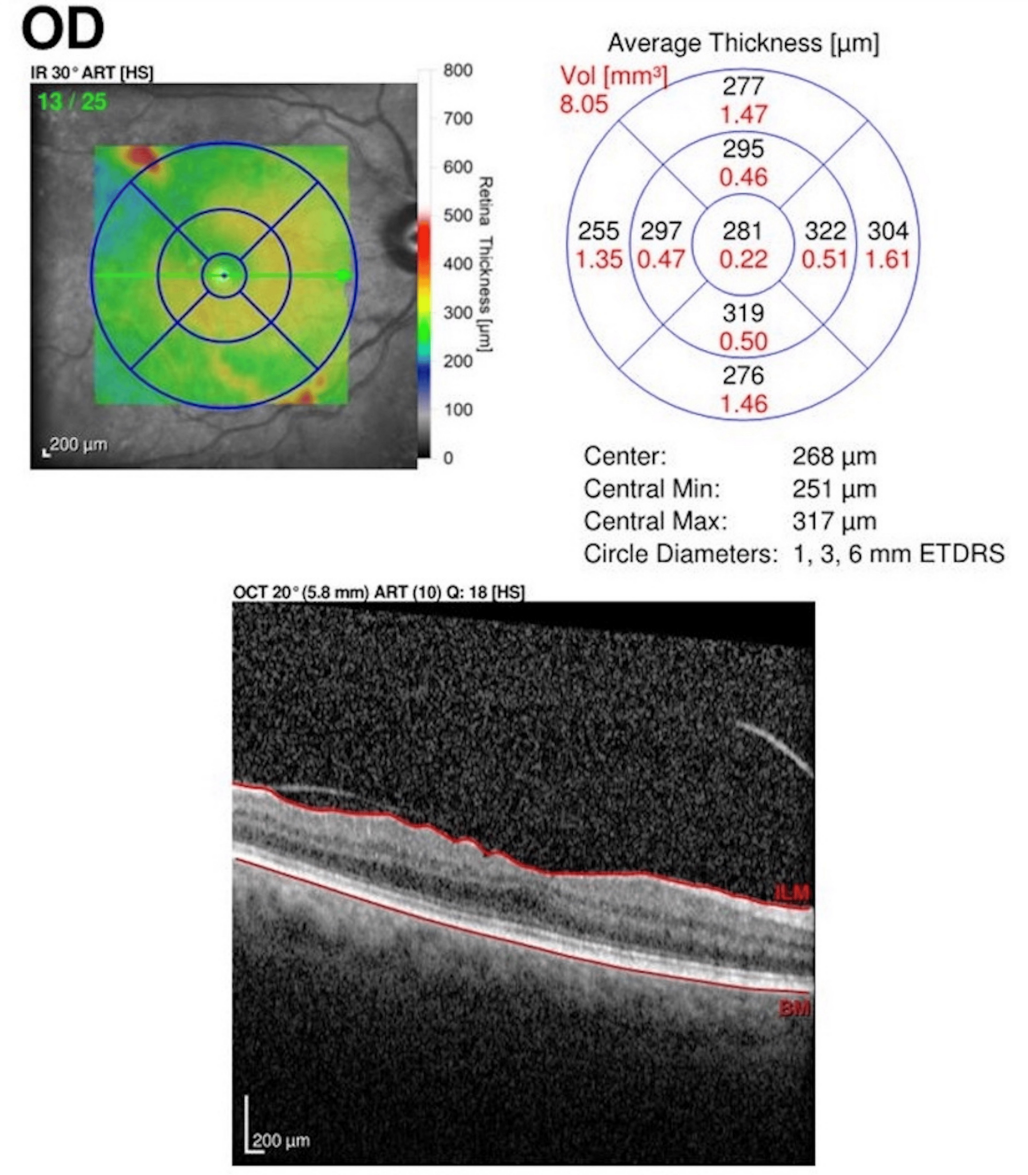
Case 2 OCT Scan at Three-Month Follow-Up OCT: optical coherence tomography

The patient is an 86-year-old Caucasian woman with a past medical history of hypertension. Presenting vision was 20/40 OD and 20/25 OS. Anterior segment examination was normal. A RAMA was noted in the right eye on the superior vascular arcade with significant exudation. OCT revealed blood products temporal to the foveola (Figure 5). The need for intravitreal injections and focal laser to the RAMA was discussed with the patient. The patient received a series of three intravitreal injections of anti-VEGF bevacizumab 1.25 mg/0.05 mL every month for three consecutive months. Focal laser (Iridex IQ 532™ Dual Port Green Laser System, Mountain View, CA), with the initial settings at 50 µm spot size, 150 mW power, and 140 ms duration, was applied one week after the anti-VEGF injection. The RAMA lesion was targeted directly. The accompanying area of thickening surrounding the RAMA was not addressed. After the focal laser, the vision improved to 20/20 in the treatment eye. At the six-week follow-up, the hemorrhage seemed to ameliorate and disperse from the foveola. At the three-month follow-up, the patient's vision in the affected eye improved to 20/30 OD. There appeared to be atrophy of the retinal pigmented epithelium and photoreceptors with shallow fibrosis sparing the foveola on OCT at the three-month visit (Figure 6).

Case 3

**Figure 7:**
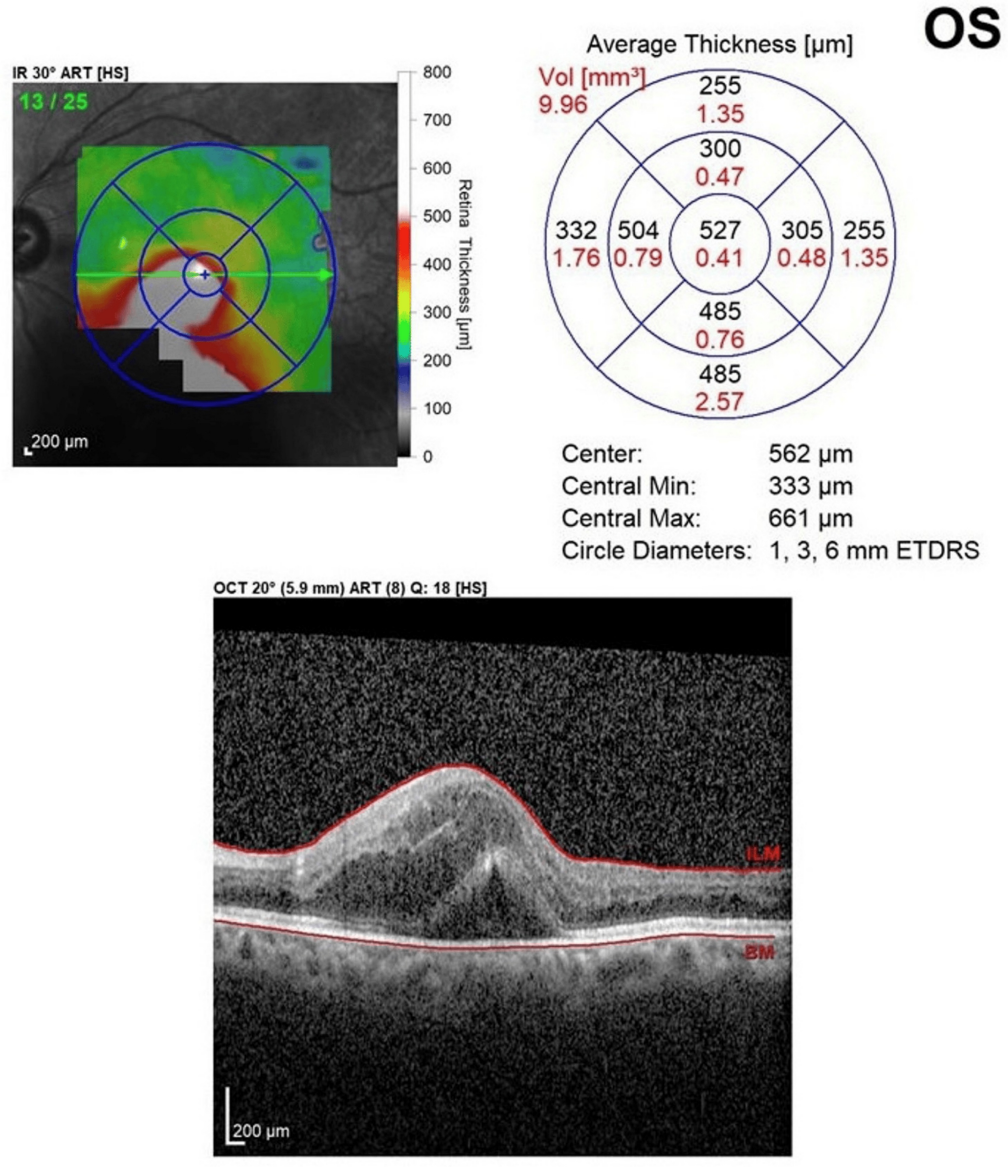
Case 3 OCT Scan Prior to Treatment OCT: optical coherence tomography

**Figure 8:**
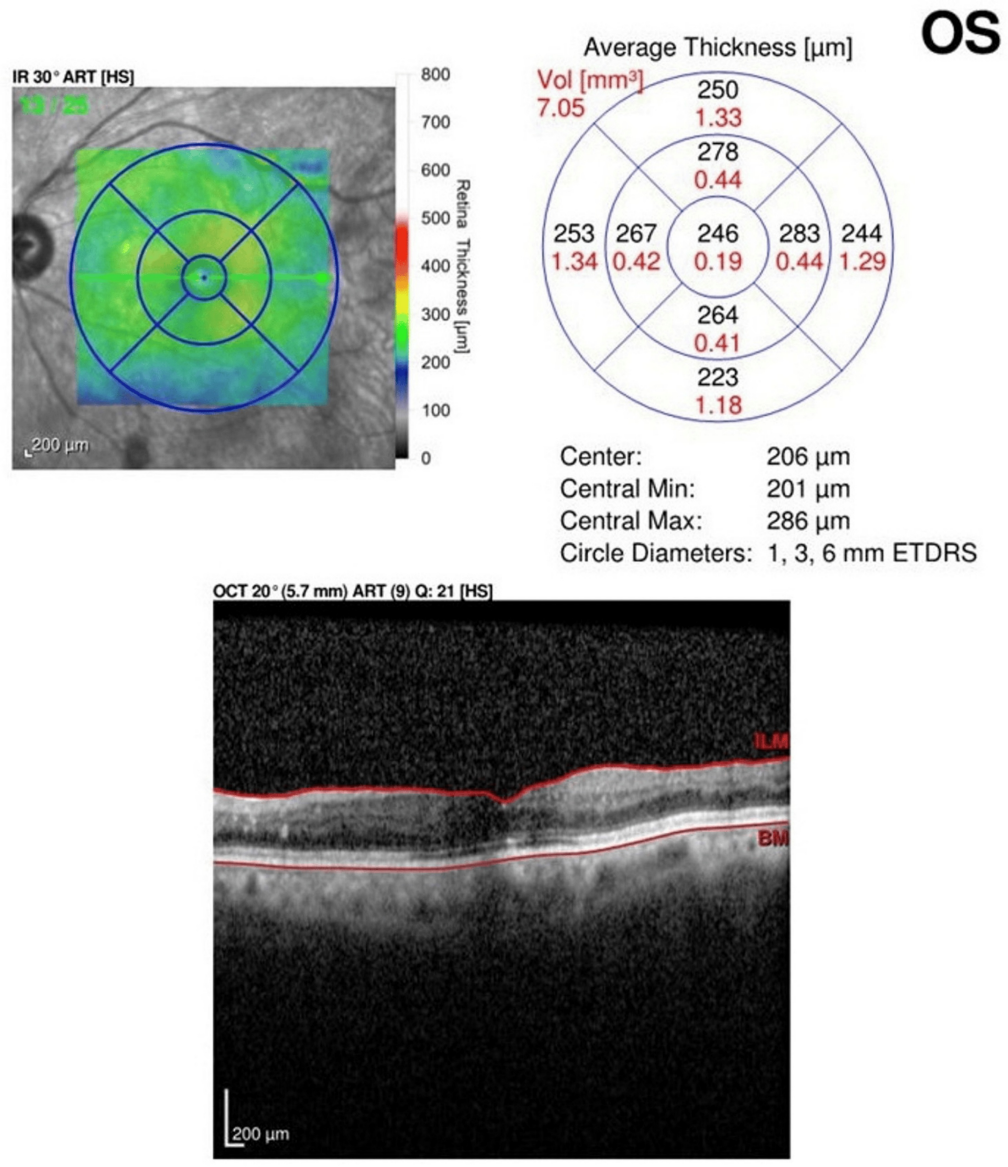
Case 3 OCT Scan at Three-Month Follow-Up OCT: optical coherence tomography

The patient is a 67-year-old Hispanic man with a past medical history of hypertension. Presenting vision was 20/30 OD and 20/80 OS. Anterior segment examination was normal. A RAMA with adjacent hemorrhage was noted superiorly, and subretinal and intraretinal fluid was noted on the foveola (Figure 7). The need for intravitreal injections and focal laser to the RAMA was discussed with the patient. The patient received a series of three intravitreal injections of anti-VEGF bevacizumab 1/25/0.05 mL every month for three consecutive months. Focal laser (Iridex IQ 532™ Dual Port Green Laser System, Mountain View, CA) was applied one week after the anti-VEGF injection with the following initial settings: 50 µm spot size, 150 mW power, and 140 ms duration. The RAMA lesion was targeted directly. The accompanying area of thickening surrounding the RAMA was not addressed. At the three-month follow-up, the vision improved to 20/50 OS. The resolution of sub- and intraretinal fluid was noted. OCT over the noted sclerosis of the lesion was performed, and the presence of perivascular fluid was not noted (Figure 8).

The tables below show the results of combined treatment in terms of central retinal thickness (CRT) and visual acuity (VA).

In our study of combined treatment, improvements in CRT and VA were demonstrated across the three analyzed cases. On average, the CRT decreased by 275.7 μm across all three cases, highlighting the therapy's overall positive impact on retinal thickness. The average presenting CRT started at 586 μm and improved to 310.3 μm among the three patients at the three-month mark (Table 1).

**Table 1:** Changes in Central Retinal Thickness (CRT) Before and After Combined Therapy

Case ID	CRT Before Treatment (µm)	CRT After Treatment (µm)	Change in CRT (µm)
Case 1	864	364	500
Case 2	367	321	46
Case 3	527	246	281
Average	586	310.3	275.7

Improvements in visual acuity were also observed in all cases. The average improvement in visual acuity across the three cases was 0.55 logarithm of the minimum angle of resolution (logMAR), indicating that the therapy generally enhanced the vision of patients. The combined average presenting visual acuity started at 1.07 logMAR and improved to 0.52 logMAR at the three-month mark (Table 2).

**Table 2:** Improvements in Visual Acuity (logMAR) Following Combined Therapy logMAR: logarithm of the minimum angle of resolution

Case ID	Visual Acuity Before (logMAR)	Visual Acuity After (logMAR)	Change in Visual Acuity (logMAR)
Case 1	2.3	1.0	1.30
Case 2	0.3	0.17	0.13
Case 3	0.6	0.39	0.21
Average	1.07	0.52	0.55

## Discussion

Overall, these outcomes suggest that the combination of focal laser therapy and intravitreal bevacizumab is effective in improving both retinal structure and visual function in patients with retinal arterial macroaneurysms (Tables 1, 2). There are concerns regarding applying a focal laser to an artery, which could potentially coagulate and consequently sclerose the retinal vessels and blood flow downstream. We believe that adjusting the laser to a 50 µm spot size, decreasing the energy power to 150 mW, and increasing the duration to 125-135 ms allow for a steady and longer application that modifies the vessel wall and decreases perforating through the vessels and Bruch's membrane. The concept was further validated by demonstrating adequate flow with fluorescein angiography in all of our patients. Fluorescein angiography was obtained in all patients post laser treatment. The angiography did not reveal filling defects distal to the RAMA in all three cases.

To date, there is no consensus regarding the appropriate treatment strategy for RAMA. In the past, multiple treatment strategies have been employed, either focal laser or intravitreal injections alone or combined with pars plana vitrectomy [1,13]. The variability in the location of the RAMA influences whether the patient is a suitable candidate for combined therapy. The location of the hemorrhage may make the patient less amenable to FL, given that a subhyaloid or sub-internal limiting membrane hemorrhage would hide the RAMA.

With respect to the use of anti-VEGF for RAMA, the paper presented by Cho et al. compared the use of anti-VEGF intravitreal injections (bevacizumab) to simple observation (control) for the treatment of RAMA in patients [1]. In the study, 24 patients were treated at the Chang Gung Memorial Hospital in Kaohsiung, Taiwan. Among the 24 patients, 25 cases of RAMA were identified, with 18 eyes affected by RAMA involving the fovea. The 18 eyes were split into two groups: 13 received anti-VEGF injections, while five were only observed. Both groups began with similar VA at the presentation. However, patients receiving anti-VEGF injections experienced significantly improved VA alongside reduced retinal swelling compared to those in the observation group. Vision improved from a logMAR of 1.52 to 0.78, and CRT decreased from 505.50 μm to 243.60 μm. The control group showed less improvement, with final vision outcomes being less favorable. These results suggest that anti-VEGF injections are highly effective for treating fovea-threatening RAMAs, leading to better long-term visual outcomes for patients. Multiple studies in our analysis, including Cho et al.'s study [1], had limitations due to their small sample sizes and retrospective design [9,10].

In another study, Chen et al. determined the efficacy of the combined laser treatment with intravitreal anti-VEGF agents. Patients underwent focal green laser photocoagulation (argon) at a wavelength of 532 nm to treat all macroaneurysms [7]. A threshold laser was administered in a confluent pattern around the macroaneurysm with a 200 μm spot size, 0.2-second duration, and 200-600 mW power until retinal whitening was observed. Subsequently, to promote thrombosis and stop hemorrhaging, a subthreshold laser was utilized directly on the RAMA. In addition, anti-VEGF was administered as one of three injection types: bevacizumab, ranibizumab, or aflibercept. From 2009 to 2010, patients with exudative RAMA were treated with laser therapy alone. From 2011 to 2016, laser therapy was combined with one of the three anti-VEGF injections within one week of the laser treatment to treat macular exudation. Thirty-five eyes from 34 patients with symptomatic RAMA were reviewed, consisting of 25 women and nine men. Among the 34 patients, 24 cases (23 patients) of hemorrhagic RAMA were identified, of which 17 were women and six were men [7]. Both the hemorrhagic and exudative RAMA groups began with different baseline visual acuities. However, after treatment, both groups showed significant improvement. In the hemorrhagic RAMA group, vision improved from a logMAR of 1.03-0.27, and all retinal and vitreous hemorrhages cleared without recurrence. In the exudative RAMA group, VA improved from a logMAR of 0.64-0.18, and CRT decreased from 330.1 μm to 236.3 μm.

These results highlight the effectiveness of combining laser therapy with anti-VEGF injections for improving visual outcomes in RAMA patients. No complications were reported, though the study had limitations due to its small sample size and retrospective design. The study also raises concerns about using direct laser photocoagulation as a treatment for RAMA, indicating it to be less favorable [7]. Studies have shown mixed results and that this may cause side effects such as scarring, retinal damage, and increased retinal exudation secondary to laser treatment [7,9,11].

## Conclusions

Combined therapy with focal argon laser and intravitreal bevacizumab has been demonstrated to be a safe and effective treatment for RAMA. RAMA is associated with physiological and anatomical alterations in the vascular profile of the affected arteriole. Structural compromise, including the breakdown of the muscular wall, results in vascular leakage and areas of non-perfusion. This process leads to an upregulation of VEGF, which further contributes to disease progression. The application of focal laser plays a critical role in stabilizing vascular architecture by promoting vessel wall remodeling and reducing the permeability of the affected arteriole. By reinforcing the structural integrity of the vessel, focal laser treatment helps to limit further leakage and non-perfusion, mitigating the pathological effects of RAMA. Intravitreal bevacizumab, an anti-VEGF agent, further complements this approach by counteracting VEGF-driven vascular permeability and neovascular complications.

Patients treated with this dual-modality approach demonstrated significant resolution of subretinal and intraretinal fluid, with notable improvements in VA. OCT imaging confirmed reduced CRT, while fluorescein angiography showed restored perfusion and vessel patency, supporting the long-term efficacy of this strategy. This combination therapy provides a targeted and durable solution for managing RAMA, improving both anatomical and functional outcomes. We recognize the intrinsic limitations of this report given its small sample size. Further prospective randomized controlled trials are to be performed to substantiate the presented data.
